# Regenerative Peripheral Nerve Interfaces Effectively Prevent Neuroma Formation After Sciatic Nerve Transection in Rats

**DOI:** 10.3389/fnmol.2022.938930

**Published:** 2022-07-07

**Authors:** Jiaqing Wu, Yajun Zhang, Xiaoyuan Zhang, Zhiyu Lin, Guangxue Li

**Affiliations:** ^1^Department of Plastic Surgery, Peking University People's Hospital, Beijing, China; ^2^Trauma Medicine Center, Peking University People's Hospital, Beijing, China; ^3^Department of Plastic Surgery, Peking University Third Hospital, Beijing, China

**Keywords:** regenerative peripheral nerve interfaces, postoperative pain, neuromas, amputation, autotomy

## Abstract

**Objective:**

The disordered growth of nerve stumps after amputation leading to the formation of neuromas is an important cause of postoperative pain in amputees. This severely affects the patients' quality of life. Regenerative peripheral nerve interfaces (RPNIs) are an emerging method for neuroma prevention, but its postoperative nerve growth and pathological changes are yet to be studied.

**Methods:**

The rat sciatic nerve transection model was used to study the effectiveness of RPNI in this experiment. The RPNI (experimental) group (*n* = 11) underwent RPNI implantation after sciatic nerve transection, while the control group (*n* = 11) only underwent sciatic nerve transection. Autotomy behavior, ultrasonography, and histopathology were observed for 2 months postoperatively.

**Results:**

Compared to the control group, the incidence and size of the neuromas formed and the incidence and extent of autotomy were significantly reduced in the RPNI group. The axon density in the stump and degree of stump fibrosis were also significantly reduced in the RPNI group.

**Conclusion:**

RPNI effectively prevented the formation of neuromas.

## Introduction

Pain is a common symptom experienced by amputees, and it is classified as postoperative pain, residual limb pain, phantom limb pain and prosthetic pain (Uustal and Meier, 2014). The incidence of residual limb pain is as high as 74% (Ehde et al., 2000), and 48.7% of these patients have evidence of neuromas (Buchheit et al., 2016). After the nerve of the amputee has been severed, the proximal nerve loses the corresponding distal nerve and innervation target, and it grows haphazardly in all directions. Neuromas are localized masses entangled with the surrounding hyperplastic fibrous connective tissue to form tumor-like structures. Neuromas can cause pain or paraesthesia when stimulated by the tearing, compressing, and stretching of the surrounding tissues (Rajput et al., 2012), and can severely impair the patients' quality of life.

In the past, the prevention and treatment methods for painful neuromas included analgesic drugs (i.e., antidepressants, anticonvulsants, opioids) (Jacobson et al., 1990; Wu et al., 2002; Robinson et al., 2004; Geary et al., 2021; Vu et al., 2022), percutaneous interventional therapies (i.e., injection of steroids, chemical ablation, cryotherapy, radiofrequency ablation, extracorporeal shockwave therapy) (Lloyd et al., 1976; Ramamurthy et al., 1989; Rasmussen et al., 1996; Fanucci et al., 2004; Markovic et al., 2008; Morgan et al., 2014), nerve conduits (Muheremu and Ao, 2015; Kubiak et al., 2018; Kang et al., 2022), surgical treatments (i.e., excision, burial, and implantation of nerve endings) and combination of multiple treatment (Azizi et al., 2012; Raisi and Mohammadi, 2019). However, the abovementioned methods have the shortcomings of unstable long-term benefits, high side effects, and a high risk of recurrence, which make the management and treatment of neuromas burdensome.

In recent years, regenerative peripheral nerve interfaces (RPNIs) (Bhashyam et al., 2021), originally designed for intelligent prosthetic control, have emerged as a treatment option for neuromas (Kung et al., 2013; Urbanchek et al., 2016; Vu et al., 2018). The severed nerve endings are implanted into free muscle grafts that target nerve regenerating axons to survive through the processes of degeneration, regeneration, revascularization, and reinnervation to achieve remodeling of the nerve-muscle junction (Svientek et al., 2020), so as to preserve nerve signals and electromyography signals (Jia et al., 2007; Langhals et al., 2014). Intelligent prosthetic control is achieved by the extraction of biological signals in RPNI (Woo et al., 2014; Irwin et al., 2016; Vu et al., 2020). During the development of this technology, it was discovered that RPNI also prevented the formation of neuromas by avoiding the disordered growth of damaged nerve axons (Woo et al., 2016; Ganesh Kumar and Kung, 2021).

Currently, RPNI has been used as a surgical procedure for the prevention of neuromas in multiple pilot clinical studies (Zimmermann, 2001; Woo et al., 2016; Hooper et al., 2020; Kubiak et al., 2022), and the efficacy of RPNI in preventing neuromas was evaluated by the use of pain scores and postoperative complications as evaluation indicators. Woo SL retrospectively analyzed 46 RPNI implantation procedures in 16 patients, and the majority of the patients reported pain relief from neuromas with great satisfaction (Woo et al., 2016). Hooper RC et al. performed 30 RPNI implantation procedures in 14 patients, and 85% of the patients were pain-free or reported improved outcomes upon long-term follow-up (Hooper et al., 2020). In the clinical study by Kubiak et al., 45 patients who received RPNI did not develop neuromas and only 51% had phantom limb pain, whereas six out of 45 patients in the control group developed neuromas and 91% had phantom limb pain. Several current clinical trials have confirmed the effectiveness of RPNI in the prevention of neuromas and phantom limb pain (Zimmermann, 2001; Pejkova et al., 2022).

However, these clinical trials have their limitations. The results of these trials were only based on the clinical outcome (i.e., the degree of pain) and it is unreasonable and inhumane to use the patients' pathological samples to explore the changes in the nerve stumps after RPNI at the cellular level because the patients' health and safety were a priority. The objective of this experiment was to observe the effect of RPNI on the regeneration process after nerve injury, using animal models and from the perspectives of behavioral studies, imaging, and pathology, so as to better understand the effectiveness and the possible mechanism of RPNI in the prevention of neuromas.

## Materials and Methods

### Ethical Approval

All the animals were purchased from Beijing Vital River Laboratory Animal Technology Co., Ltd. The experimental protocol was approved by the animal ethics committee of the Peking University People's Hospital, approval number 2020PHE050, and the experiments were carried out in the Laboratory Animal Unit of the Peking University People's Hospital. All experiments were designed according to the Animal Research: Reporting of *in vivo* Experiments (ARRIVE) guidelines.

### Animals and Surgical Procedures

**Figure 1:**
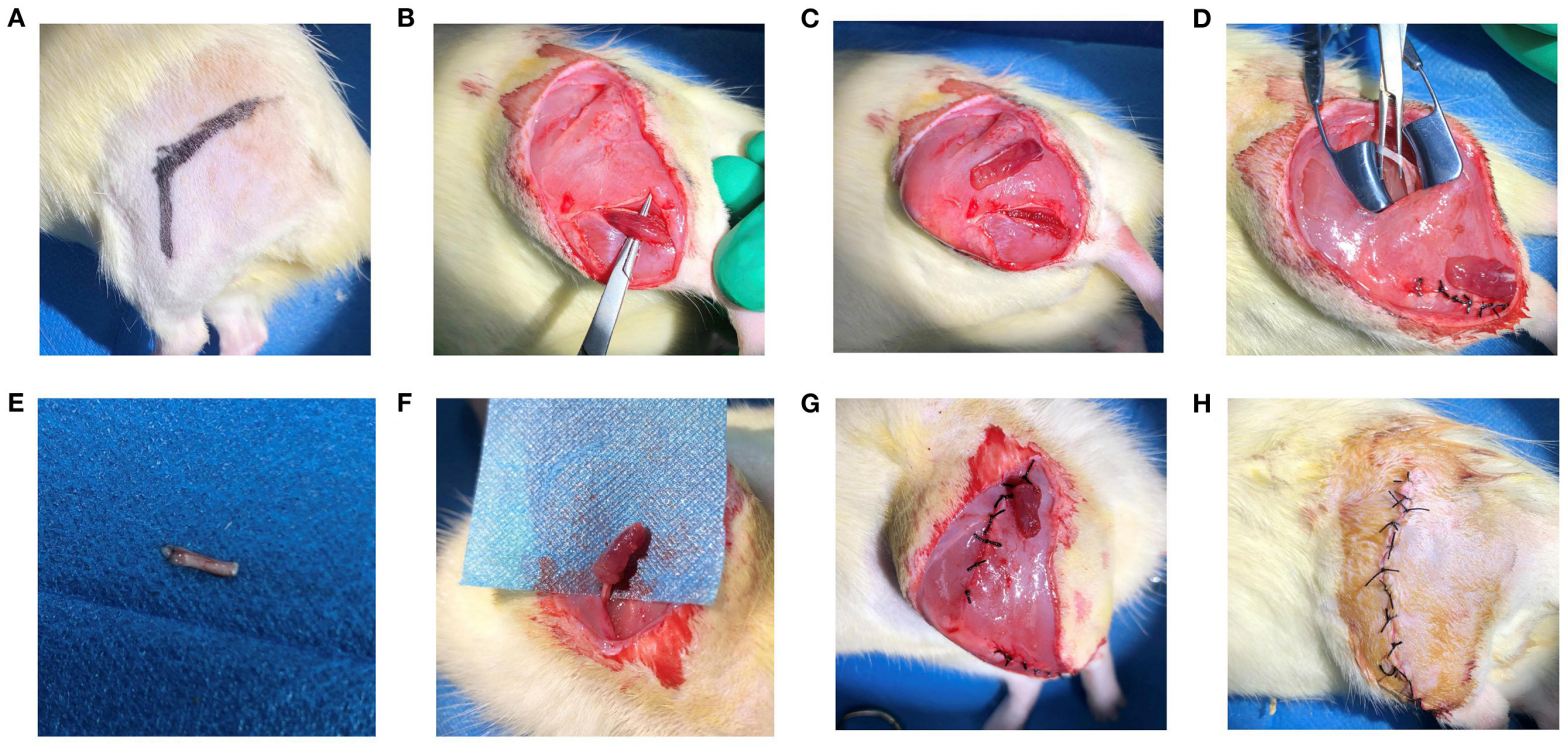
**(A)** Confirm the surgical approach by exposing the lower limb skin of the rat. **(B)** Expose the extensor digitorum longus of the rat. **(C)** Cut off and acquire the appropriate size extensor digitorum longus. **(D)** Expose the sciatic nerve of the rat. **(E)** Transect the sciatic nerve of the rat. **(F)** Fix the nerve adventitia on the obtained free extensor digitorum longus muscle, and suture the muscle to wrap the nerve. **(G)** Suture the muscle space and fix the RPNI model under the skin. **(H)** Suture the skin and establish the RPNI model.

Twenty-two Sprague Dawley rats (6 weeks-old, 200–300 g in bodyweight) were used in this study. The rats were randomly divided into the RPNI and control groups (*n* = 11/group). All the experimental animals were anesthetized by inhalation of 3% isoflurane (500 mL/min) (RWD Life Science, Shenzhen, China). The process surgical operation of RPNI is shown in Figure 1. In a sterile operating room, a longitudinal incision was made in the posterolateral aspect of the rat's thigh and extended to the posterolateral aspect of the calf. The skin and subcutaneous tissue were incised in turn to reveal a musculocutaneous perforator (retrogluteal musculocutaneous perforator). The sciatic nerve was quickly exposed, by entering from the intermuscular septum between the biceps femoris and the semitendinosus muscle close to the anterior side of this perforator. The sciatic nerve was transected and 1 cm was resected at the distal end to avoid reconnection of the nerve. A 7-0 suture was used to mark the branch of the posterior gluteal nerve under the operating microscope as a marker for sampling at the end of the experiment. Subsequently, the tibialis anterior muscle was incised along the anterolateral side of the calf to expose the extensor digitorum longus (EDL) muscle, which was fully dissociated from the popliteal fossa and the foot and then transected from the tendon. The freed EDL was harvested. Then, the EDL was placed at the proximal end of the sciatic nerve, and the epineurium and the muscle membrane were sutured with four stitches of 9-0 suture. The 9-0 suture was longitudinally sutured to the muscle to wrap the nerve ending and complete the establishment of a RPNI. The established RPNI was placed in the lower outer thigh with its position fixed subcutaneously. The control group did not receive any special treatment after sciatic nerve transection. The incision was closed with a 4-0 silk thread and the skin was sutured to close the wound of all the rats.

### Behavioral Observation of Self-Mutilation (Autotomy)

Using a double-blind method, the autotomy scores of each group of rats were observed and recorded thrice a week by two researchers. The autotomy scores were quantified according to the modified scale by Wall et al. (1979), i.e., 1 point for the absence of two or more toenails per limb, with a maximum of 1 point per limb; and 1 point for missing half of each toe (distal and proximal), up to 10 points per limb.

### Ultrasonography

The Toshiba Apolio 500 color ultrasonic diagnostic instrument (Toshiba, Tokyo, Japan) with line array probe and frequency of 5–14.0 MHz was used. Two months postoperation, the rats in each group were fully anesthetized, and the skin of the inspection region was prepared. The projection region of the sciatic nerve on the surgical side of the rat was fully exposed, and two-dimensional ultrasound and color Doppler flow imaging were performed to observe the size, shape, echo, and blood supply of the sciatic nerve tumor. Two researchers measured separately to reduce subjective errors.

### Specimen Preparation

All animals were euthanised 2 months postoperation by inhalation of carbon dioxide. The carbon dioxide replacement rate was set to 30% per minute. The skin and muscle were opened layer by layer at the surgical scar, and the 7-0 suture marking was identified for positioning. The proximal nerve stump was collected and fixed in 4% paraformaldehyde at 4°C.

### Histological Analysis

After dehydration in ethanol, clearing with xylene, and embedding in paraffin, the distal end of the nerve specimen (i.e., the surgically severed end) was transected into sections of 5 μm thickness. Each specimen was subjected to α-SMA immunohistochemical staining to assess the degree and extent of tissue fibrosis and NF-200 immunofluorescence staining to detect axonal density. The following steps were followed for α-SMA staining: the sections were deparaffinised in water, heat-induced antigen retrieval with EDTA, incubated with 3% hydrogen peroxide for 25 min, and blocked with 5% goat serum for 30 min. Rabbit anti-α-SMA antibody (1:2,000, Cat# 14395-1-AP, RRID:AB_2223009, Proteintech, Beijing, China) was added and incubated at 4°C overnight, followed by incubation with biotinylated anti-rabbit IgG secondary antibody (1:200, Cat# ZB-2010, ZSGB-BIO, Beijing, China) at room temperature for 1 h, diaminobenzidine (DAB) staining was performed under microscopy, and the nuclei were counterstained with haematoxylin, dehydrated and mounted. The following steps were followed for NF-200 immunofluorescence staining: the sections were deparaffinised in water and subjected to antigen retrieval with EDTA. After blocking with 5% goat serum (Solarbio) for 30 min, the sections were incubated with mouse anti-NF-200 antibody (1:200, Cat# N5389, RRID: AB_260781, Sigma, St. Louis, MO, USA) overnight at 4°C, and stored for 2 h at room temperature with Alexa Fluor 594 anti-mouse IgG (1:200, Cat#ZF-0513, ZSGB-BIO). The nuclei were stained with 4′,6-diamidino-2-phenylindole (Sigma).

All images were captured by a Leica DM4 B microscope (Leica, Wetzlar, Germany). Four fields of each pathological were randomly selected and analyzed by Image-Pro Plus 6.0 software (Media Cybernetics, Rockville, MD, USA). The percentage of α-SMA positive staining area was calculated as the α-SMA positive staining area / the total image area × 100. The density of axons was defined as the number of axons within an area of a field.

### Statistical Analysis

All numerical data are presented as the mean ± standard deviation (SD). The experimental results were analyzed with SPSS 22.0 software (IBM, Armonk, NY, USA) and the Student's *t*-test was used. Differences were statistically significant at *P* < 0.05.

## Results

### RPNI Significantly Reduced Autotomy Behavior in Rats

**Figure 2:**
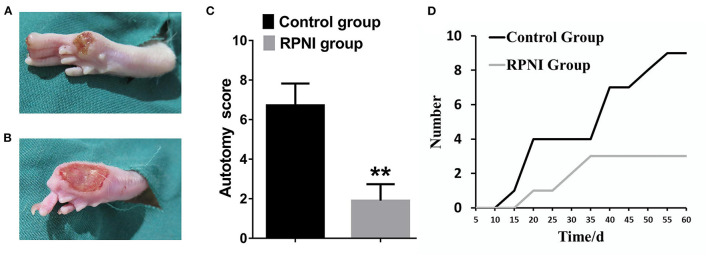
**(A)** Representative pictures of autotomy behavior of rats in the RPNI group. **(B)** Representative pictures of autotomy behavior of rats in the control group. **(C)** Autotomy score of the control group and autotomy score of the RPNI group. (**P < 0.01). **(D)** Autotomy in the control group appeared earlier than that in the RPNI group, and the number of rats with autotomy increased with time.

Autotomy was observed in three mice in the RPNI group and in nine mice in the control group at 2 months after the surgery of RPNI. At about 15 days after operation, autotomy began to appear in some rats, and the time in the control group was earlier than that in the PRNI group. The autotomy behavior is shown in Figure 2, and the autotomy score of the RPNI group was much lower than that of the control group (*P* < 0.01). RPNI significantly reduced the autotomy behavior in rats.

### RPNI Significantly Reduced the Degree of Nerve Stump Hyperplasia

**Figure 3:**
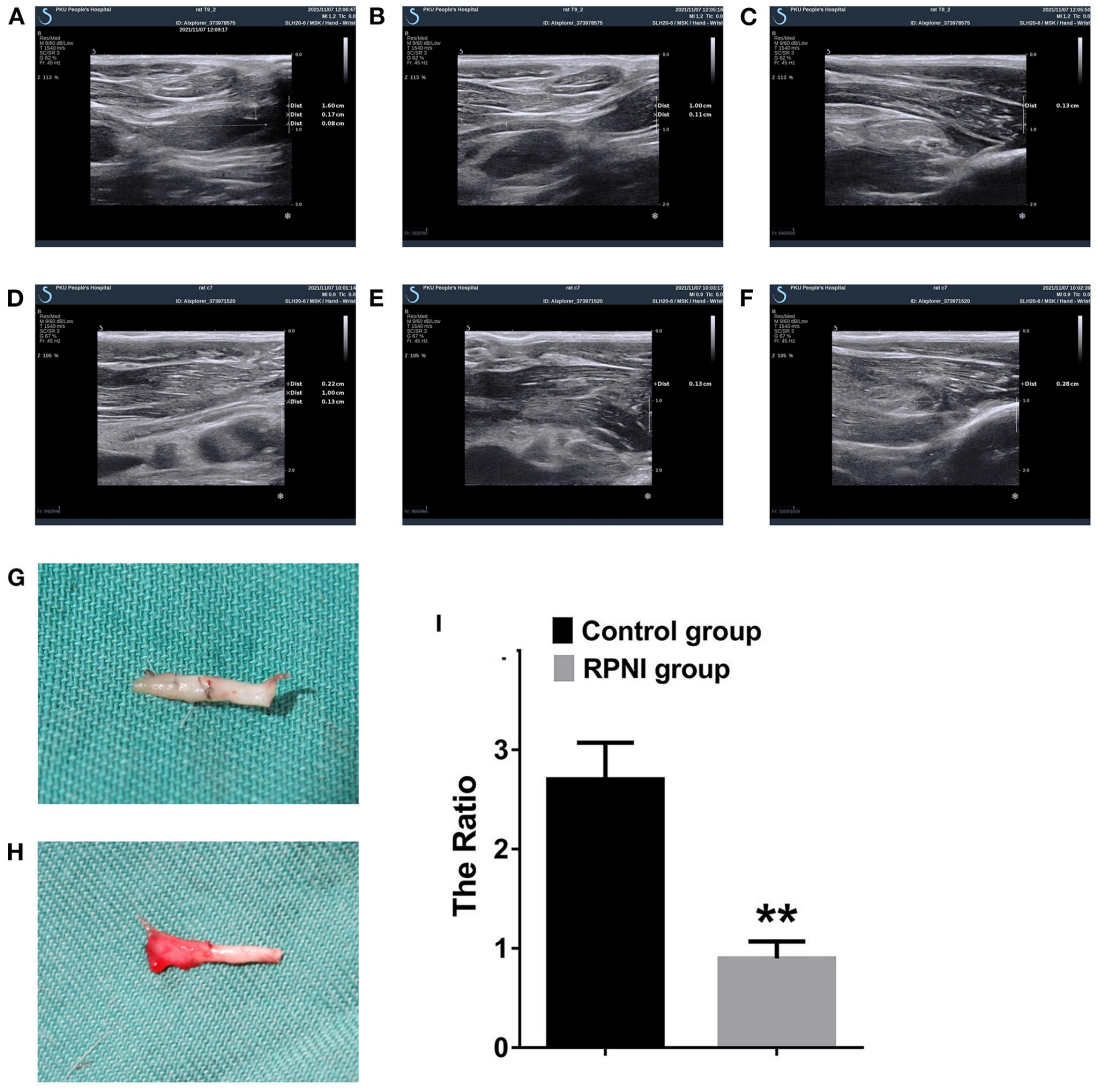
Ultrasound images of rat nerve endings at 8 weeks postoperation. **(A)** Longitudinal section of RPNI rat along the course of the nerve (+ indicates the muscle used to wrap the nerve in the establishment of RPNI, × indicates the distal end of the nerve ending, which is the severed end, ^
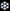
^ indicates the proximal end of the nerve, i.e., relatively normal nerve tissue). It is observed that the regeneration of nerve stumps in RPNI rats was insignificant, and there was no obvious nerve enlargement. **(B)** Proximal nerve ending in a RPNI rat. **(C)** Distal nerve ending of a RPNI rat. **(D)** The longitudinal section of a control group rat along the course of the nerve, without muscle wrapping, and showed that the nerve was significantly thickened. **(E)** Proximal nerve ending of a rat from the control group. **(F)** Distal nerve ending of a rat from the control group. **(G)** The morphology of the nerve of the RPNI group. There was no obvious expanded neuroma at the end of the nerve. **(H)** The morphology of the nerve of the control group. Expanded neuroma can be observed at the end of the nerve. **(I)** The ratio of the cross-sectional size of the nerve stump to the normal nerve tissue at the proximal end of the nerve endings is estimated by the product of the long diameter and the short diameter of the cross section. There are significant differences between the control group and the RPNI group (**P < 0.01).

As shown in Figure 3, the nerve stumps of the RPNI rats did not increase significantly, while the nerve stumps of the rats in the control group increased significantly and tended to form neuromas. In addition, the ratio of the cross-sectional size of the nerve stump to the normal nerve tissue at the proximal end of the nerve endings in the RPNI group was significantly lower than that in the control group (*P* < 0.01), which indicated that RPNI significantly reduced the degree of regeneration after nerve injury and achieved the goal of preventing neuromas.

### RPNI Inhibited the Regenerative of Axons After Sciatic Nerve Transection

**Figure 4:**
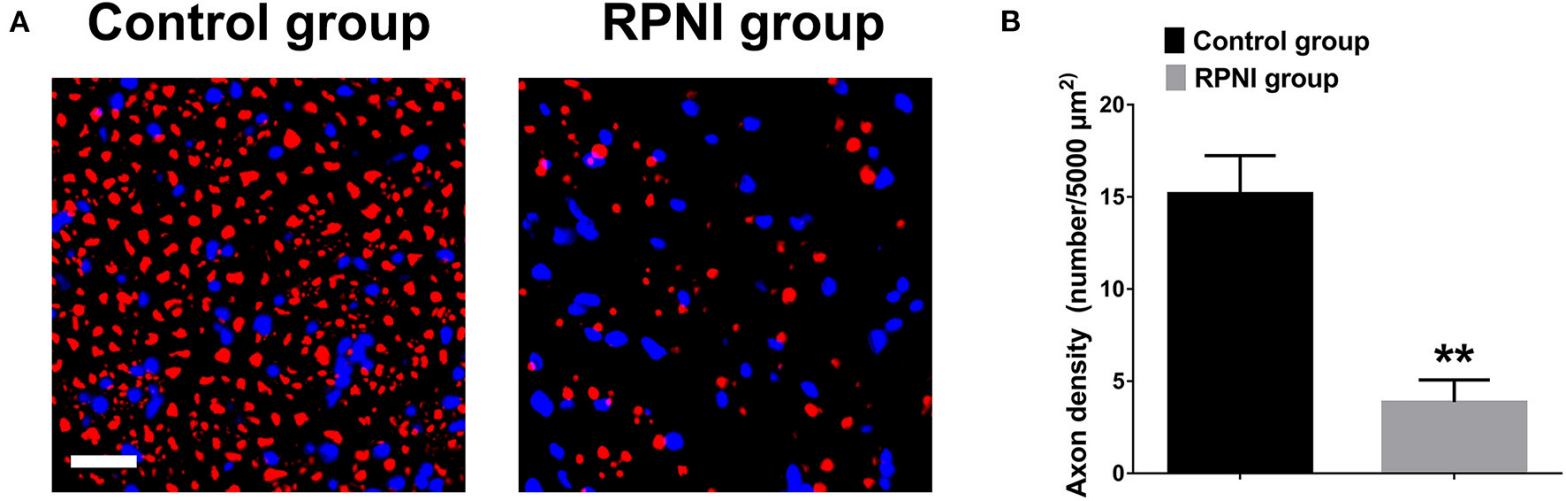
Regenerative peripheral nerve interfaces (RPNI) significantly inhibited axonal regeneration after sciatic nerve transection. **(A)** NF200 immunofluorescence staining of cross sections of the proximal nerve stump. The fluorescence indicator used was Alexa Fluor 594 for NF200 (red). Nuclei are shown in blue. Scale bars: 20 μm. **(B)** Quantitative results of the density of regenerated axons (**P < 0.01).

Neuromas are formed by the regenerative and disorderly growing axonal after nerve injury. RPNI inhibited the regenerate of axons after sciatic nerve transection. As shown in Figure 4, the axonal density revealed by NF-200 immunofluorescence staining of the RPNI group was significantly lower than that of the control group (*P* < 0.01).

### RPNI Effectively Reduced the Degree of Neural Fibrosis in the Sciatic Nerve Stump

**Figure 5:**
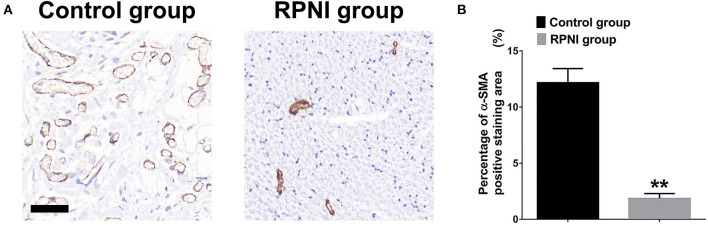
Regenerative peripheral nerve interfaces (RPNI) effectively reduced the degree of neural fibrosis in the sciatic nerve stump. **(A)** α-SMA immunohistochemical staining (brown) of cross sections of the proximal nerve stump. Scale bars: 40 μm. **(B)** Quantitative results of the percentage of α-SMA positive staining area (**P < 0.01).

RPNI inhibited the proliferation of fibroblasts after nerve injury to a certain extent and reduce the entanglement of axons with surrounding fibrous connective tissue which may be beneficial to reduce inflammation around nerve terminals (Zwetsloot et al., 2012; Lieber and Ward, 2013). As shown in Figure 5, α-SMA immunohistochemistry revealed that the positive area in the control group was larger (*P* < 0.01).

## Discussion

When peripheral nerves are damaged, the nerves that have lost their distal innervated muscles grow in a disordered manner and become entangled with the surrounding tissues to form neuromas, which causes pain. Most of the previous prevention and treatment methods for neuromas were ineffective, but the development of RPNI provided a new direction for the resolution of painful neuromas.

At present, the effectiveness of RPNI in preventing the formation of neuromas is generally recognized (Loewenstein et al., 2022), and its utilization rate is increasing gradually. The research on the postoperative safety of RPNI (Lans et al., 2021) and more innovative surgical methods [c-RPNI (Svientek et al., 2020), TMRpni (Svientek et al., 2022), MC-RPNI (Kurlander et al., 2020), etc.] are also underway. This study attempted to investigate the efficacy of RPNI in preventing neuromas from multiple levels through a sciatic nerve amputation model in rats.

Autotomy behavior in rats is often used to evaluate the degree of nerve pain after peripheral nerve injury (Zimmermann, 2001). Marcol et al. (2011) applied microcrystalline chitosan to prevent neuromas, while Pi et al. (2022) used myelin-associated glycoproteins in combination with chitin catheters to prevent the formation of neuromas in their experiments, and used the autotomy behavior of rats as one of the evaluation indicators. Two months following the operation performed on the rats in both the groups, the autotomy behavior of the RPNI group was significantly milder than that of the control group (regardless of the number of rats with autotomy behavior or the degree of autotomy). Since the autotomy behavior of the rats might be affected by environmental, psychological, and other factors, we ensured that the living environment, diet, age, and bodyweight of the two groups of rats were the same, with the only the surgical procedures performed being different. To a large extent, RPNI is considered to be effective in relieving pain after sciatic nerve transection.

Ultrasound imaging is an intuitive way to observe live neuromas. It can accurately measure the thickening of the nerve endings without injuring the rat, so that the experimental data is not damaged during the sampling process. The ratio of the transverse diameter of the nerve stump to the proximal transverse diameter of the rat nerve endings in the RPNI group was smaller in the ultrasound images, which proved that RPNI effectively inhibited the disordered growth and tumourigenesis of nerves.

Svientek et al. found that 3 months after RPNI, muscle revascularization and nerve remodeling were completed (Kubiak et al., 2021), and neuromuscular junctions in the muscle were successfully observed (Svientek et al., 2020). This finding demonstrated that the muscles involved in RPNI wrapping of nerve stumps achieved muscle-nerve junction remodeling during vascular remodeling, and revealed the possibility of RPNI being used as a muscle-nerve interface to extract biological signals. When we dynamically observed the ultrasound images of individual rats, we discovered an interesting phenomenon that confirmed this finding. The growth of the muscle (EDL) used to wrap the nerve in RPNI showed a trend of atrophy followed by surviving growth. At the same time, the proximal sciatic nerve was stimulated with electrodes when the specimen was taken, and the contractile movement of the EDL could be observed. This shows that RPNI forms a complete pathway of brain—spinal cord—peripheral motor nerve—muscle.

NF-200 immunofluorescence staining also confirmed that RPNI effectively reduced irregular axonal growth after nerve injury. NF-200 (neurofilament protein-200) is an important substance that provides structural support to the axons and regulates the diameter of axons. It is arranged in parallel with axons and can reflect the number and growth of axons. In this experiment, NF-200 immunofluorescence staining was used to evaluate the number of axons after nerve injury, and the axonal density of the RPNI group was significantly lower than that of the control group, which proved that RPNI effectively inhibited the regenerative axons which result in the formation of the neuroma after the nerve injury.

In order to further evaluate the degree of neural fibrosis in the two groups, α-smooth muscle actin (α-SMA) immunohistochemical staining was performed. The positive rate of α-SMA was lower in the RPNI group, which indicated that RPNI inhibited fibroblast proliferation and attenuated stump fibrosis after nerve injury to a certain extent. Fibrosis and tangle of regenerated axons and fibrous connective tissue are the pathological basis of neuroma formation. Fibrosis is also related to the activation of a variety of inflammatory pathways and the regulation of gene expression (Zwetsloot et al., 2012; Klingberg et al., 2013; Lieber and Ward, 2013).

Inevitably, this experiment has its limitations. Firstly, the evaluation of pain in rats was relatively limited, and the observation of pain markers such as substance P was lacking. Secondly, there was a lack of more microscopic structural observations. Painful neuromas were thought to be related to various factors such as local inflammation and cytokines, increased Na^+^ channel density, and disordered growth of unmyelinated nerve fiber buds after nerve injury (Vu et al., 2020). This experiment did not involve deeper and broader research into the electrophysiology, inflammatory factors such as TNF-α, and observation under electron microscope. Thirdly, there was no comparison with other neuroma prevention procedures, and the superiority of RPNI in comparison with conventional neuroma prevention procedures and the application of various new materials that has not yet been proven. These require further research.

## Conclusion

RPNI prevented the formation of neuromas by inducing physiological self-limitation, limiting the regeneration of injured axons, reducing the random and irregular arrangement of regenerated nerve fibers, and preserving the possibility of extracting biological signals through the reconstruction of the neuromuscular junction. It provides more possibilities for the prevention and treatment of postoperative complications of amputees. The potential mechanism and application of RPNI still needs further research and development.

## Data Availability Statement

The original contributions presented in the study are included in the article/supplementary material, further inquiries can be directed to the corresponding author/s.

## Ethics Statement

The animal study was reviewed and approved by the Animal Ethics Committee of the Peking University People's Hospital.

## Author Contributions

GL and YZ contributed to the conception of the study. GL, JW, XZ, and ZL performed the experiment. JW and YZ contributed significantly to analysis and manuscript preparation. All authors contributed to the article and approved the submitted version.

## Funding

Project (2020YBC12) supported by Peking University Education Big Data Project. Project (RDY2020-04 and RDG2021-04) supported by Peking University People's Hospital Research and Development Funds.

## Conflict of Interest

The authors declare that the research was conducted in the absence of any commercial or financial relationships that could be construed as a potential conflict of interest.

## Publisher's Note

All claims expressed in this article are solely those of the authors and do not necessarily represent those of their affiliated organizations, or those of the publisher, the editors and the reviewers. Any product that may be evaluated in this article, or claim that may be made by its manufacturer, is not guaranteed or endorsed by the publisher.
